# Chemical Evaluation of Energy Dispersive X-ray Spectroscopy Analysis of Different Failing Dental Implant Surfaces: A Comparative Clinical Trial

**DOI:** 10.3390/ma14040986

**Published:** 2021-02-19

**Authors:** Berceste Guler, Ahu Uraz, Hasan Hatipoğlu, Mehmet Yalım

**Affiliations:** 1 Department of Periodontology, Faculty of Dentistry, Kütahya Health Sciences University, Kütahya 43100, Turkey; hasan.hatipoglu@ksbu.edu.tr; 2 Department of Periodontology, Faculty of Dentistry, Gazi University, Ankara 06500, Turkey; ahuuraz@gazi.edu.tr (A.U.); myalim@gazi.edu.tr (M.Y.)

**Keywords:** dental implant, surface properties, photoelectron spectroscopy, peri-implantitis

## Abstract

The aim of the present study is to compare two different implant surface chemistries of failing dental implants. Sixteen patients (mean age: 52 ± 8.27 with eight females and eight males) and 34 implants were included in the study. Group-I implants consisted of a blasted/etched surface with a final process surface, while Group-II implants consisted of the sandblasted acid etching (SLA) method. The chemical surface analysis was performed by the energy dispersive X-ray spectroscopy (EDX) method from coronal, middle, and apical parts of each implant. Titanium (Ti) element values were found to be 20.22 ± 15.7 at.% in Group I and 33.96 ± 13.62 at.% in Group-II in the middle of the dental implants. Aluminum (Al) element values were found to be 0.01 ± 0.002 in Group-I and 0.17 ± 0.28 at.% in Group II in the middle of the dental implants, and statistically significant differences were found between the groups for the Al and Ti elements in the middle of the dental implants (*p* < 0.05). There was a statistically significant difference for the Ti, Al, O, Ca, Fe, P, and Mg elements in the coronal, middle, and apical parts of the implants in the intragroup evaluation (*p* < 0.05). It is reported that different parts of the implants affected by peri-implant inflammation show different surface chemistries, from coronal to apical, but there is no difference in the implants with different surfaces.

## 1. Introduction

Peri-implantitis is an inflammatory disease characterized by alveolar bone loss and increased peri-implant pocket depth [1]. Studies have shown that surface characteristics such as roughness, surface chemistry, and topography increase osseointegration; however, the roughness of the surface makes implant surface decontamination difficult for peri-implantitis treatment. Studies have reported that the risk of peri-implantitis changes for different dental implants with different surface properties [2,3].

Studies have been reported to explain alveolar bone loss due to factors such as implant surface chemistry or corrosion in the occurrence of peri-implantitis. In a study, it was reported that titanium (Ti) is a reactive material, and a nano-thick layer of titanium oxide (TiO_2_) forms on the surface of the dental implant in contact with air or water [4]; this oxide layer increases the biocompatibility, material-bone interface compatibility, and corrosion resistance of the implant [5]. In a hypothesis presented by Mouhyi et al., it was reported that the formation of an acidic electrochemical environment due to excessive mechanical stress and bacterial biofilm causes the destruction of the oxide layer on the surface of the dental implant and that peri-implant fluids in direct contact with the Ti can be expected to affect this oxide layer [6]. In another hypothesis proposed by Fretwurst, it was claimed that the Ti element released from dental implants could be expected to lead to host tissue response, resulting in peri-implant bone destruction [7]. A review was conducted showing that particles released from the implant surface due to corrosion or mechanical-chemical surface processes cause an inflammatory reaction and thus alveolar bone loss [8].

There is limited evidence of the relationship between implant surface features and design as risk factors for peri-implantitis and marginal bone loss after functional loading. In previous studies, marginal bone loss was reported to be higher in implants with rough surfaces than in implants with moderately rough surfaces [9,10,11]. Another study also reported that peri-implantitis occurred in rough surface implants, and no peri-implantitis cases were recorded in machined implants [12]. However, in a 20 year follow-up study, no difference was found between the machined surface and the rough surface implants in terms of peri-implantitis [13].

Carbon (C), nitrogen (N), calcium (Ca), phosphorus (P), chlorine (Cl), sulfur (S), sodium (Na), and silicon (Si) were detected in addition to a titanium oxide layer on the implant surfaces [12,13]. It is hypothesized that these elements are released as part of an inflammatory response to the chemically degraded implant surfaces; this inflammatory response affects the healing process and provokes the dissolution of Ti [6]. In another study, it was reported that examination of the properties of the implant surface and the chemistry of failing implants would be valuable for understanding the changes caused by peri-implantitis on the implant surface [7].

The hypothesis of this study is that dental implants with different surface properties contain different chemical elements in the areas affected by peri-implantitis. This study aimed to comparatively evaluate the changing implant surface chemical properties due to peri-implantitis in dental implants subjected to different chemical surfaces.

## 2. Materials and Methods

### 2.1. Study Population and Eligibility Criteria

This study was designed as a prospective, parallel arm clinical trial, and the study design was approved by the Dumlupınar University Clinical Research Ethics Committee (Research No. 2017-2/5). The study was carried out between January 2017 (first enrollment) and April 2019 (last enrollment) in the Department of Periodontology, Faculty of Dentistry, Kütahya Health Sciences University and Gazi University. The research was conducted in accordance with the Declaration of Helsinki of 1975, revised in 2013. All patients were informed and signed consent forms.

Patients were included with the following criteria: systemically healthy, no radiotherapy or chemotherapy, no temporomandibular joint problems, psychological acceptance of surgical and prosthetic treatment, good oral hygiene, no medication for bone metabolism disorder (especially bisphosphonate), and no pregnancy or lactation.

Patients who applied to the periodontology departments in both centers who had dental implants were included in the study according to certain criteria after clinical and radiological examination and the Dental Implants Health Scale [14]. According to the scale, dental implant removal indications are as follows: (a) pain in function; (b) mobility: a tweezer was fixed at the buccal or palatinal side of the crown; the other tweezer was moved horizontally (buccolingual); and the presence and degree of mobility were evaluated according to the Miller classification [15]; (c) radiographic bone loss that was higher than ½ of the implant length; (d) the persistent presence of pus after phase I treatment.

Dental implants with the following criteria were excluded from the study: (1) peri-implant pocket depth of 5 mm or less after phase I treatment; (2) vertical bone defects; (3) periapical infection around the implant area; (4) antibiotic usage for at last 6 months.

Dental implants were examined in two parts according to their surface characteristics. Group I implant consisted of a blasted/etched surface with a final process surface, while Group II implants consisted of the sandblasted acid etching (SLA) method. Group I blasted and final process surfaces were Legacy II (Implant Direct, Thousand Oaks, CA, USA) and Bicon Integra CP (Bicon systems, Boston, MA, USA) dental implant brands, while Group II implants consisted of Straumann BL Tapered (Straumann Holding, Basel, Switzerland), XiveR S (Dentsply Sirona Implants, Mannheim, Germany), DTI (TUBITAK, Gebze, Kocaeli, Turkey), Neo Implants (Alpha Biotec Implantology, Washington, DC, USA), MIS C1 (Divident, Yehudah, Israel), and AstraTech (Denstply Sirona, Gothenburg, Sweden) implants with SLA surfaces.

### 2.2. Clinical and Radiographical Measurements

All clinical measurements were recorded using the Williams-type periodontal probe, and participants received the phase I treatment and oral hygiene instructions. Patients were revaluated after four weeks of phase I periodontal treatment, and the data obtained from the peri-implant region with extraction indication were as follows as outcome measurements: (1) periodontal pocket depth (PD); the distance between the gingival margin and the bottom of a pocket; (2) clinical attachment level (CAL); the distance from the neck of the implant and the bottom of the pocket; (3) implant length: the distance between the dental implant apical point and implant shoulder. Implant length was measured by a periodontal probe after the dental implant was extracted, and surface chemical analysis was performed. Clinical measurements were recorded using a periodontal probe from four points around the peri-implant region (mesiobuccal, distobuccal, mesiolingual, distolingual).

Radiographic measurements such as marginal bone loss and implant length were obtained by a digital software program [16] (Mediadent Software, The Dental Imaging Company, London, UK). Radiographic bone loss (RBL) was measured on periapical radiographs. Bone loss level was measured with software as the distance from the implant shoulder and the first bone-implant contact. For each implant, one RBL value was calculated as the mean of the mesial and distal values [17]. The implant length was evaluated as the distance between the implant shoulder and the most apical point of the implant in the software program. The bone loss/implant length ratio was measured on periapical radiographs, and values were recorded as percentages.

Occlusal trauma: Occlusion, bite papers, and primary contacts were evaluated for primary evaluation. Bruxism and myofascial pain were evaluated for chronic occlusal trauma.

Primary outcome measurements were implant surface chemical analysis. Secondary outcome measurements were the relation with chemical analysis and periodontal pocket depth, clinical attachment level, and implant length.

### 2.3. Retrieval Procedure for the Dental Implants

After local anesthesia was administered, it was removed using a stainless-steel forceps by rotating counterclockwise. To avoid damaging the surface on which the chemical analysis of the dental implants would be performed, it was ensured that the end of the forceps did not exceed the implant-abutment junction [13]. After removal of the dental implant, blood was removed from the surface of the implant with saline; the implant was then dehydrated with gradually ethanol solutions ranging from 70 to 99 % alcohol [13], dried at room temperature, and placed in a sterile plastic box.

### 2.4. Scanning Electron Microscopy and Energy Dispersive X-ray Spectrometry Analysis

All dental implants were taken from their storage boxes as delivered. They were handled with tweezers.

**Figure 1:**
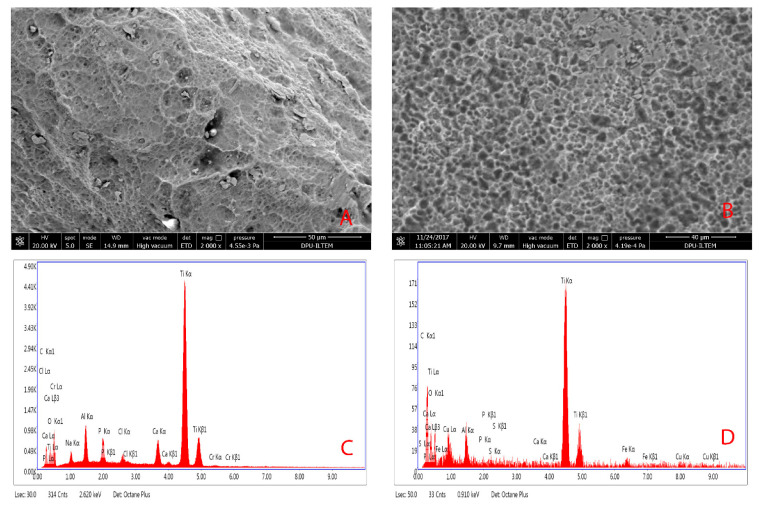
Scanning electron microscopy images of a failing dental implant: (**A**) Group I implant surface at ×2000 magnification; (**B**) Group II implant surface at ×2000 magnification; (**C**) EDX analysis diagram of Group I; (**D**) EDX analysis diagram of Group II.

Images of the samples were taken using a field emission scanning electron microscope Nova^TM^ NANOSEM 650 (FEI Company, Hillsboro, OR, USA) at the same working distance ranging between 6 and 15 mm, an acceleration voltage of 20 kV, a beam current of 650 pA, and an acquisition time of 150 live seconds. All images of dental implants were also morphologically evaluated using a calibrated scanning electron microscope (SEM) at ×250 and ×2000 (Figure 1). The samples were placed on carbon discs and carbon plaster for fixation and then placed inside the microscope chamber under vacuum conditions.

**Figure 2:**
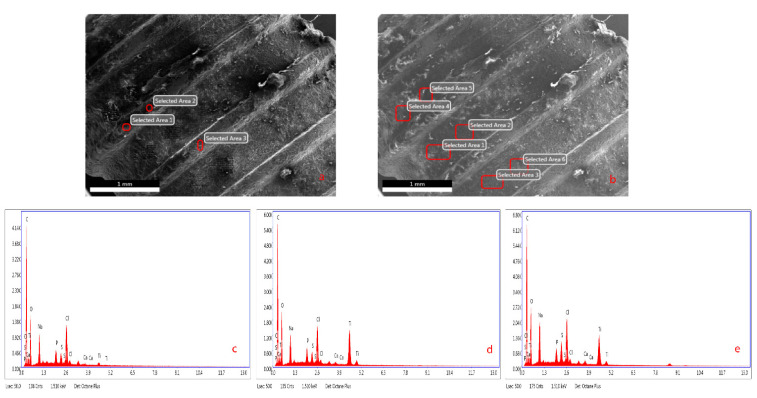
(**a**,**b**) SEM images on the top, valley, and flank parts of fixtures; (**c**) EDX analysis of the top parts; (**d**) EDX analysis of the valley parts; (**e**) EDX analysis of the flank parts of fixtures.

Energy dispersive X-ray spectrometry (EDX) analyses of all implants were performed on the coronal, middle, and apical regions of the implants. Additionally, chemical surface analyses with EDX were performed on the top, valley, and flank parts of the screw of each implant in these three regions. (Figure 2)

### 2.5. Statistical Analysis

The data were analyzed using the software program SPSS [18] for Windows v. 20.0. All parameters were analyzed by the Kolmogorov–Smirnov test for normality distribution. Continuous variables were expressed as mean ± SD and categorical variables as numbers and percentages. The groups were compared using the Mann–Whitney U test for continuous variables. Normality distributions were evaluated using the Shapiro–Wilk test for analysis within the group. Since Ti, O, C, and N are parametric data, the one-way ANOVA test was used for analysis; the Kruskal–Wallis test used for the K, Al, Ca, Cu, Cr, Fe, S, P, and Mg elements was non-parametric. Multivariate stepwise linear regression analyses were conducted using all three different models for periodontal pocket depth; the coronal, middle, and apical regions of dental implants as the dependent variables. For all the equations, the independent variables were those with *p*-values < 0.05 in univariate analyses (for the coronal region of implants, Mg, N, Al; for the middle region of implants; for the apical region of implants S, Al, P, N). A *p*-value less than 0.05 was considered statistically significant.

## 3. Results

A total of 16 patients, 8 females and 8 males, and 34 implants were included in the study. The mean age of the included patients was 52 ± 8.27 years, between 33 to 62 years.

### 3.1. Demographic Data

The survival time of the implants ranged from 18 months to 14 years, and the mean value was 4.94 ± 3.18 years. Maxillary and mandibular molar regions were the most common locations from which the implants were retrieved. Dental implants were examined in two parts according to their surface properties. Group I consisted of surfaces obtained by blasted and etched surfaces with a final process (n = 9), while Group II were the sand blasted acid etching (SLA) method (n = 25). The dental implant brand distribution is shown in Table 1.

The dental implants were evaluated for dental implant health according to clinical measurements and using the Dental Implant Health Scale [14]. Indications for retrieval of dental implants were pain in function 44.4% in Group I and 72% in Group II, pus formation recorded as 55.5% in Group I and 76% in Group II, and mobility seen in 8% in Group II. The ratio of radiographic bone loss to implant length was measured as 0.55 ± 0.39% in Group I and 0.47 ± 0.33% in Group II. (Table 2).

The mean values of the peri-implant clinical measurements are given in Table 3. The mean PD value was 5.39 ± 2.25 mm for Group I and 6.12 ± 1.76mm for Group II; the mean CAL value was 5.73 ± 2.7 mm for Group I and 6.3 ± 1.65 mm for Group II; and there were no statistically significant differences between the groups. The mean implant length was found to be 7.89 ± 2.24 mm for Group I and 9.32 ± 1.43 mm for Group II, and there were statistically significant differences between the groups (*p* = 0.037) (Table 2).

### 3.2. Surface Chemistry Analysis

The chemical surfaces of the coronal, middle, and apical regions of all dental implants were analyzed between the groups, and the results are shown in Table 3. According to the results, the Ti, O, and C elements were detected on all implant surfaces. The weighted elements were Al, Ca, N, and P, and the Fe, Cr, Mg, Cu, K, and S elements were found in very small amounts and on a small number of implant surfaces.

There were statistically significant differences in the middle region of dental implants for the Al and Ti elements as percentages of the atomic number of the atoms (at.%). Ti element values were found to be 20.22 ± 15.7 in Group I and 33.96 ± 13.62 at.% in Group II in the middle of the dental implants. Al element values were found to be 0.01 ± 0.002 at.% in Group I and 0.17 ± 0.28 at.% in Group II in the middle of the dental implants, and there were statistically significant differences found between the groups for the Al and Ti elements in middle of the dental implants (*p* < 0.05) (Table 3).

Intragroup analyses were done from the coronal, apical, and middle regions of the implants and are shown in Table 4. In Group I, a statistically significant difference was found between the implant parts for the Ti, O, C, Al, Ca, Fe, P, and Mg elements. In Group II, a statistically significant difference was found between the implant parts for the Ti, O, N, Al, Ca, S, Fe, P, Mg, and Cu elements (Table 3).

Multivariate stepwise linear regression analysis of the periodontal pocket depth scores for the dental implants’ coronal, middle, and apical models is presented in Table 4. For the coronal periodontal pocket depth model, the independent variables in the equation were Mg-coronal, N-coronal, and Al-coronal. The coefficient of determination R^2^ of the subjective age was 0.407, indicating that these three factors can explain 41% of all coronal periodontal pocket depth model variations. For the middle periodontal pocket depth model, the only independent variable was Al-middle and the coefficient of determination.

R^2^ was 0.169, indicating Al-middle can explain 16 % of all variations of the middle model of periodontal pocket depth. Lastly, for the apical periodontal pocket depth model, the independent variables in the equation were S-apical, Al-apical, P-apical, and N-apical. The coefficient of determination R^2^ of the apical model was 0.556, indicating that these four factors can explain 55 % of all apical periodontal pocket depth model variations (Table 4).

## 4. Discussion

In the study, a statistically significant difference was found in terms of the Ti and Al values in the evaluation of two different implant surface chemistries, but no significant difference was found in terms of other elements between the groups. Otherwise, intragroup surface chemistry evaluation between the different parts of the implants was found to be statistically different. Although the chemistry of the affected surfaces due to peri-implant inflammation was mostly similar in implants with different implant surface properties, statistically significant different chemical values were found between different regions of the implant affected by peri-implant inflammation in intra-group evaluations. These findings may also support that implant coronal to apical surfaces affected by peri-implant inflammation show different chemical compositions.

Recently, a new definition of osseointegration was reported [2]: “osseointegration is a foreign body reaction where interfacial bone is formed as a defense reaction to shield off the implant from the tissues.” Excessive hydrogen peroxide (H_2_O_2_) production occurs against implant foreign bodies, and the TiO_2_ layer on the surface thickens. Oxygen radicals integrate into the implant surface, and the thickening of the TiO_2_ layer allows integration of Ca and P ions from the alveolar bone to the TiO_2_ surface [19,20]. The protective oxide layer on the metal structures ensures that the ions released due to corrosion are kept at a very low level and there is no non-corrosive metal in contact with body fluids [21]. In a study, peri-implant bone and implant surfaces were evaluated by EDX analysis using human cadavers, and Ca and P were found in the peri-implant alveolar bone tissue, while only Ti was found on the implant surface [22]. The chemical analysis performed by Shibli et al. on failed implants suggested that elements such as C, Ca, Na, and P result from the absorption of dissolved ions naturally formed from body fluids [19]. It is known that bone resorption and bone remodeling occur in bone tissue in response to inflammation [23]. In the present study, Ti and O levels increased on both implant surfaces from coronal to apical. However, like the above-mentioned study, it was seen that the Ca and P values also increased. It can be said that with the increase in TiO_2_ thickness, Ca and P elements on the implant surface increase, and ions in the alveolar bone are the origin.

In some commercial implant systems, different implant surface modification techniques are used to increase osseointegration and reduce corrosion resistance. Sand blasting and acid etching are the most used techniques, and aluminum oxide (Al_2_O_3_) particulates can be used [24]. Sulfuric acid, hydrochloric acid, hydrofluoric acid, or combinations thereof can also be used for acid etching [25]. Ca, P, and Al as the dominant elements in the previously reported studies; therefore, it has been suggested that modification techniques could be responsible for some of these elements detected on the implant surfaces [26]. In this study, it is reported that peri-implant pocket depth is related to the Mg, N, and Al elements in the coronal region, to Al in the middle region, and to S, Al, P, and N in the apical region on SLA surfaces. The relationship between Al in all regions of the dental implants and the depth of the peri-implant pocket can be associated with Al_2_O_3_ powders in the process part of the SLA surface. Although the Al element was detected in Group I in the regression analysis performed, no statistically significant relationship was found with the peri-implant pocket depth.

Studies have reported that microorganisms such as Porphyromonas gingivalis (P. gingivalis), which causes periodontal disease, also produce volatile sulfur compounds [27] in acute infections of peri-implantitis with pus, especially red complex microorganisms [28]. Studies have also reported that especially P. gingivalis and red complex microorganisms are the predominant periodontal pathogens of failing dental implants [26,29]. Studies have reported that a large proportion of C and a lower proportion of N, Cl, and S may be relevant in some instances due to contamination of the implant during the construction process [30,31]. In a study, EDX was evaluated using various removal methods on the biofilm layer on the SLA surface of Ti discs placed in the mouth. The C, Si, S, and Ti elements were detected in the control group where only air and water were applied. It has been reported that the source of the C, S, and Si elements can be saliva and biofilm [32]. In the present study, the C element increased in both groups from the coronal to the apical region; however, statistical significance in Group I was found, and there was no statistical significance difference in Group II. The S element concentrations were found to have a statistically significance higher concentration in the coronal area compared to the apical region in both groups, independent of the implant surface properties; however, there were no statistically significant differences between the two different surface characteristics. The sulfur component present on the implant surface may be related to the end products of the microorganisms.

An in vitro study reported that microorganism and bacterial end products cause damage to the titanium oxide layer and change the chemical surfaces of the implants [25]. It has been found that multiple microorganisms such as Aggregatibacter actinomycetemcomitans (A.actinomycetemcomitans), Streptococcus salivarius (S. salivarius), Streptococcus sanguinis (S. sanguinis), and Streptococcus mutans (S. mutans) cause organic acid release from sugar metabolism and cause the corrosion of metals in contact with peri-implant crevicular fluid by making an acidic environment [28]. In some studies, the titanium oxide layer was covered with a C-rich contamination layer, and N, Ca, P, Cl, S, Na, and Si materials have also been reported [21,22]. In another study, it was suggested that the C, N, Cl, S, and Ca elements were found on some dental implant surfaces and may have been absorbed during the preparation of the implant surface [19]. A study evaluated the chemical analysis and surface roughness of different implant surfaces and reported that alumina oxide abrasives from sandblasting were found on the implant surfaces as a contaminant [33]. Dental implants made of metal alloys release potentially harmful ions such as Cr, Co, and Al due to corrosion at the bone-implant interface, and the accumulation of metallic ions can be considered as one of the reasons for implant failure [34]. In this study, especially the SLA surface, increased peri-implant pocket depth and the Al element were related. Furthermore, while the Cu element was not found in blasted surface dental implants, the Cu element was detected in the chemical analysis of SLA surfaces. The Cr element was detected on both implant surfaces, and no difference was found between the groups. When the Al element shows a significant difference in terms of peri-implantitis in the analysis between groups and is associated with the peri-implant pocket depth, the potential for long-term peri-implantitis risk should be evaluated, especially on SLA surfaces.

In another study, it was reported that the Si and P elements could be by-products of Ti production, and that Ca and Na could come from body fluids. It was also reported that Si and C could pass through plastic gloves [35]. Furthermore, it is also thought that elements such as Fe, Mg, Cu, and K, which are detected in small amounts, may be due to contamination during modification of the implant surface or during implant extraction or examination. It is known that the iron ion in hemoglobin is used for the growth of microorganisms such as A.actinomycetemcomitans in relation to Fe [36]. Another study reported that after functional loading and non-osseointegration, when failing dental implants were evaluated by EDX analysis, only Ti and Fe were detected on the surfaces of the implants; however, 38 implants had a mechanical indication for extraction, and the implants had no functional loading [37].

Different ions such as Cl in the saliva can cause metallic corrosion [21]. In a study, a biofilm layer was placed on Ti discs with an SLA surface, and the discs were washed with sterile saline, so NaCl was not included in the EDX analysis results [32]. In this study, although the Na and Cl elements were detected on the chemical surfaces of all implants, they were excluded from the study since they would not provide accurate information about NaCl because the dental implants were extracted and washed with sterile saline.

In a study, microbiological analyses of failed dental implant surfaces revealed that organic materials were observed in different regions, and EDX analysis revealed that titanium oxide and the C, O, N, Na, Ca, and P elements were detected on the surface [37]. A study was reported the chemical analysis of nine different implant systems with X-ray photoelectron spectroscopy (XPS) analysis; N, C, and O were the most common elements, and the source may be the atmosphere; it has been reported that the C ratio is particularly high on the TiUnite surface roughened by the anodization method and Osseospeed surfaces treated with hydrofluoric acid [33]. In this study, it was found that the C ratio in the coronal region was low in both groups and increased towards the apical region. Considering that the high ratio of TiO and the C element is related to osseointegration, it can be considered that the change in the implant chemical surface due to peri-implantitis may cause implant failure.

In a case report, the Ti, O, Al, N, C, F, Ca, Zn, and S elements were detected on the surface of a Ti6Al4V dental implant removed 18 years later by XPS analysis [38]. In a study, it was reported that the Ti, O, N, and Al elements were detected by deep region analysis of the implants, and it was stated that the basic elements of this implant could be N and Al in addition to Ti and O [21]. Annunziata et al. reported that commercially produced Ti can have different degrees of purity and contain elements such as O, C, and Fe as intermediate elements [39]. In a study presented by Ehrenfest et al., the identification cards of the surface properties of dental implants were evaluated, and it was reported that different pollutants were detected during the modification on many surfaces [40]. In the study, it was also mentioned that different ratios of elements in dental implants with the SLA surface obtained with the same technique were the reason for the pollution [40]. A case report was conducted showing that the N element may originate from the atmosphere [38], but in our study, the N element was found at high rates in the coronal region in both groups and could not be detected in the apical regions. Again, in the regression analysis performed, it was found that the N element was associated with the depth of the peri-implant pocket. In our study, it is thought that the N element may be associated with peri-implant inflammation. In our study, dental implants with different roughnesses and topographies were included in both implant surface groups. For this reason, the presence of different brands of implants in the groups makes it difficult to detect and evaluate elements previously specified as impurities in implant identity.

The peri-implant surface properties are important for removing the biofilm layer to resolve peri-implant inflammation, and the surface roughness and chemical composition of the implant surface can have a negative effect on plaque deposition [41]. A study compared dual acid etched surfaces and hybrid acid etched surfaces in terms of peri-implantitis risk; however, there was no statistically significant difference found between the two surfaces [42]. A study compared dual etched, titanium plasma spray, hydroxyapatite coated surfaces, and turned surfaces, and all surfaces were equally susceptible to ligature-induced peri-implantitis [43,44]. In another study, it was reported that the surfaces obtained by anodization had a greater risk of developing peri-implantitis than blasted or acid etching and blasted surfaces [45]. Different implant brands blasted, blasted with acid etching, and treated with the anodization method were evaluated in terms of surface cleanliness, and it was reported by EDX analysis that organic and inorganic contaminants such as iron, magnesium, and aluminum were found in some brands and that these contaminants are structures that disrupt the continuity of the implant surface features [46]. Furthermore, different elements such as Al, P, and N have been reported as inorganic-organic contaminations in some implant brands [46]. Especially, in the SLA group in our study, peri-implant pocket depth was found to be correlated with the Al, Mg, N, and S elements. In the present study, different elements were determined according to the articles in which implant surface identities were evaluated before, and according to our results, there were no differences between implants with different surfaces.

As a limitation, two different implant surfaces were compared in the study, but despite having the same intragroup chemical surface features, the dental implants had different macro and micro designs. Furthermore, it would be better to have resonance frequency analysis measurements done before dental implant extraction. Comparing dental implants of the same brand and surface chemistry may be better in terms of the elimination of confounding factors. When evaluating implant surface chemistry, evaluating the sterile packed surface chemistry of each implant brand and the surface chemistry after peri-implantitis may be effective for evaluating the impact of peri-implant infection on the implant surface. The length of time the implant surface is exposed to peri-implant inflammation can also affect the study results. Therefore, it will be beneficial to study the different survival times of implants with the same implant surface feature and the effect on the implant’s chemical surface.

## 5. Conclusions

In conclusion, contaminants such as Al, Mg, N, and S are associated with peri-implant pocket depth, especially on SLA surfaces; it is reported that the chemical surface composition of the implant from the coronal to the apical region is associated with peri-implant infection, but there were no difference chemical elements between the different implant surfaces. In further studies, the relationship between the dental implant chemical surface and peri-implant inflammation and pocket depth can be better demonstrated in dental implants of the same brand.

## Figures and Tables

**Table 1:** Demographic data of the dental implants.

Dental Implant Location	(n = 34 Dental Implants)
Right-left maxillary molar	12 (35.29%)
Right-left mandibulary molar	18 (52.94%)
Right-left maxillary incisors	1 (2.94%)
Right-left mandibulary incisors	3 (8.82%)
Implant survival year (mean ± SD; min-max year)	4.94 ± 3.38 (1.5–14)
Implant commercial name and surface structure	
Xive S (Dentsply Sirona Implants, Mannheim, Germany)	2
Neo Implants (Alpha Biotec Implantology, Washington, DC, USA)	7
Legacy II (ImplantDirect, Thousand Oaks, CA, USA)	7
DTI (TUBITAK, Gebze, Kocaeli, Turkey)	4
AstraTech (Denstply Sirona, Mölndal, Sweden)	3
Bicon Integra CP (Bicon systems, Boston, MA, USA)	2
MIS C1 (Divident, Yehudah, Israel)	7
Straumann BL Tapered (Straumann Holding, Basel, Switzerland)	2

**Table 2:** Comparison of periodontal measurements and Dental Implant Health Scale parameters.

Clinical Measurements	Group I (n = 9) Mean ± SD	Group II (n = 25) Mean ± SD	*p*-Values
PD	5.39 ± 2.25	6.12 ± 1.76	0.280
CAL	5.73 ± 2.7	6.3 ± 1.65	0.216
Implant Length (mm)	7.89 ± 2.24	9.32 ± 1.43	0.037
Pus	55.5% (5/9)	76.0% (19/25)	0.355
Functional Pain	44.4% (4/9)	72.0% (18/25)	0.237
Radiographic Bone Loss/Implant Length	0.55 ± 0.39	0.47 ± 0.33	0.584
Occlusal Trauma %	44.4% (4/9)	24% (6/25)	0.092
Mobility (Miller Class 2)	0	8.0% (2/25)	-

PI: Plaque Index, GI: Gingival Index, PD: periodontal pocket Depth, GR: gingival recession, CAL: clinical attachment level, KGW: keratinized gingival width, BOP: bleeding on probing. The Mann–Whitney U Test was performed for statistical analysis. Statistical significance was *p* < 0.05.

**Table 3:** The comparative chemical surface analysis of different regions of dental implant surfaces as atomic weight % between the groups and intragroup.

	Group I				Group II				*p*-Values
	Coronal *	Middle **	Apical ***	*p*-Values	Coronal *	Middle **	Apical ***	*p*-Values	
Ti	6.17 ± 6.48	20.22 ± 15.69	23.26 ± 19.1	0.049	3.59 ± 4.25	33.96 ± 13.62	33.36 ± 17.47	0.000	* 0.22 ** 0.02 *** 0.23
O	39.36 ± 19.4	3.8 ± 2.5	26.29 ± 3.0	0.000	30.6 ± 11.17	3.67 ± 4.31	26.29 ± 9.7	0.000	* 0.15 ** 0.69 *** 0.84
C	19.77 ± 5.01	21.3 ± 9.79	40.9 ± 22.08	0.007	24.9 ± 9.7	22.9 ± 8.5	28.7 ± 16.65	0.247	* 0.14 ** 1.00 *** 0.15
N	27.86 ± 21.24	0	0	0.661	36.85 ± 18.93	7.32 ± 3.29	0	0.000	* 0.20 ** - *** 0.13
Al	0.03 ± 0.06	0	4.51 ± 2.65	0.000	0.15 ± 0.25	0.16 ± 0.27	4.94 ± 3.8	0.000	* 0.20 ** 0.02 *** 0.70
Ca	0.02 ± 0.07	6.6 ± 16.01	2.09 ± 2.57	0.001	0.04 ± 0.17	2.64 ± 2.32	3.6 ± 3.2	0.000	* 0.84 ** 0.14 *** 0.16
S	1.3 ± 2.87	0.26 ± 0.44	0.12	0.264	1.32 ± 0.24	0.11 ± 0.15	0.21 ± 0.18	0.001	* 0.33 ** 0.81 *** 0.16
K	3.03 ± 3.76	0	0.4	0.853	3.99 ± 3.91	0.4	0.27	0.955	* 0.59 ** -*** 0.32
Fe	0.1 ± 0.16	1.38	0.42	0.066	0.04 ± 0.08	0.7 ± 0.18	0.36 ± 0.06	0.001	* 0.24 ** 0.22 *** 0.22
Cr	0.5 ± 0.98	0.34	0	0.590	0.62 ± 1.08	0.14 ± 0.13	0.27 ± 0.07	0.537	* 0.66 ** 0.31 *** -
P	0	0.69 ± 0.93	1.7 ± 1.56	0.000	0.17 ± 0.08	1.3 ± 1.45	1.77 ± 1.65	0.000	* 0.55 ** 0.39 *** 0.96
Mg	0	0	0.3	0.003	0.1 ± 0.42	0	0.55	0.001	* 0.39 ** - *** 0.32
Cu	0	0	0	-	0.46 ± 0.23	0.57 ± 0.68	1.17 ± 0.5	0.000	* 0.55 ** - *** -

The Mann–Whitney-U test was performed for between groups (*p* value). One-way ANOVA and Kruskal–Wallis tests were used for intragroup comparison (*p* value). *p*- and *p*-values were 0.05 for statistical significance. *p* values indicated the significance value for between the groups: * coronal, ** middle, *** apical.

**Table 4:** Multiple step linear regression analysis of elements affecting the relationship between peri-implant pocket depth and implant surface chemical analysis in Group II.

Peri-Implant PD Elements	B	SE	β	t	*p*-Value
Peri-implant PD-Coronal *					
Mg	12.678	3.734	0.49	3.395	0.002
N	0.197	0.071	0.397	2.777	0.009
Al	0.138	0.055	0.361	2.536	0.017
Peri-implant PD-Middle **					
Al	0.201	0.079	0.411	2.548	0.016
Peri-implant PD-Apical ***					
S	6.065	1.31	0.617	4.629	0.000
Al	0.345	0.08	0.604	4.307	0.000
P	0.14	0.051	0.355	2.725	0.011
N	0.367	0.173	0.311	2.122	0.042

* F = 6.863, R = 0.638, R^2^ = 0.407; ** F = 6.49, R = 0.411, R^2^ = 0.169; *** F = 9.088, R = 0.746, R^2^ = 0.556; Β: partial regression coefficient; β: standard regression coefficient; SE: standard error; Mg: magnesium; N: nitrogen; Al: aluminum; S: sulfur; P: phosphorus.

## Data Availability

Data can be shared by contacting the corresponding author.
